# Virtual reality as a method of cognitive training of processing speed, working memory, and sustained attention in persons with acquired brain injury: a protocol for a randomized controlled trial

**DOI:** 10.1186/s13063-024-08178-7

**Published:** 2024-05-22

**Authors:** T. Johansen, M. Matre, M. Løvstad, A. Lund, A. C. Martinsen, A. Olsen, F. Becker, C. Brunborg, J. Ponsford, J. Spikman, D. Neumann, S. Tornås

**Affiliations:** 1 Department of Research, Sunnaas Rehabilitation Hospital, Nesodden, Norway; 2 Department of Psychology, Faculty of Social Sciences, University of Oslo, Oslo, Norway; 3 Department of Occupational Therapy, Institute of Rehabilitation Science and Health Technology, Faculty of Health Sciences, Oslo Metropolitan University, Oslo, Norway; 4 Department of Life Sciences and Health, Faculty of Health Sciences, Oslo Metropolitan University, Oslo, Norway; 5 Department of Psychology, Norwegian University of Science and Technology, Trondheim, Norway; 6 Clinic of Rehabilitation, St. Olavs Hospital, Trondheim University Hospital, Trondheim, Norway; 7 NorHEAD - Norwegian Centre for Headache Research, Trondheim, Norway; 8 Department of Physical Medicine and Rehabilitation, University of Oslo, Oslo, Norway; 9 Oslo Centre for Biostatistics & Epidemiology, Oslo University Hospital, Oslo, Norway; 10 Turner Institute for Brain and Mental Health, School of Psychological Sciences, Monash University, Clayton, Australia; 11 Monash-Epworth Rehabilitation Research Centre, Epworth Healthcare, Richmond, Australia; 12 Department of Neurology, Subdepartment of Neuropsychology, University of Groningen, University Medical Center, Groningen, The Netherlands; 13 Department of Physical Medicine and Rehabilitation, Indiana University School of Medicine, Indianapolis, USA

**Keywords:** Virtual reality, VR, Cognitive rehabilitation, Cognitive training, ABI, Stroke, Traumatic brain injury

## Abstract

**Background:**

Acquired brain injury (ABI) often leads to persisting somatic, cognitive, and social impairments. Cognitive impairments of processing speed, sustained attention, and working memory are frequently reported and may negatively affect activities of daily living and quality of life. Rehabilitation efforts aiming to retrain these cognitive functions have often consisted of computerized training programs. However, few studies have demonstrated effects that transfer beyond the trained tasks. There is a growing optimism regarding the potential usefulness of virtual reality (VR) in cognitive rehabilitation. The research literature is sparse, and existing studies are characterized by considerable methodological weaknesses. There is also a lack of knowledge about the acceptance and tolerability of VR as an intervention method for people with ABI. The present study aims to investigate whether playing a commercially available VR game is effective in training cognitive functions after ABI and to explore if the possible effects transfer into everyday functioning.

**Methods:**

One hundred participants (18–65 years), with a verified ABI, impairments of processing speed/attention, and/or working memory, and a minimum of 12 months post injury will be recruited. Participants with severe aphasia, apraxia, visual neglect, epilepsy, and severe mental illness will be excluded.

Participants will be randomized into two parallel groups: (1) an intervention group playing a commercial VR game taxing processing speed, working memory, and sustained attention; (2) an active control group receiving psychoeducation regarding compensatory strategies, and general cognitive training tasks such as crossword puzzles or sudoku. The intervention period is 5 weeks. The VR group will be asked to train at home for 30 min 5 days per week.

Each participant will be assessed at baseline with neuropsychological tests and questionnaires, after the end of the intervention (5 weeks), and 16 weeks after baseline. After the end of the intervention period, focus group interviews will be conducted with 10 of the participants in the intervention group, in order to investigate acceptance and tolerability of VR as a training method.

**Discussion:**

This study will contribute to improve understanding of how VR is tolerated and experienced by the ABI population. If proven effective, the study can contribute to new rehabilitation methods that persons with ABI can utilize in a home setting, after the post-acute rehabilitation has ended.

**Supplementary Information:**

The online version contains supplementary material available at 10.1186/s13063-024-08178-7.

## Introduction

### Background

Acquired brain injury (ABI) affects an estimated 200 per 100,000 of the global population [1], and represents a severe health issue which often leads to life-long somatic, cognitive, emotional and social impairments. Many persons with ABI need intensive, interdisciplinary rehabilitation efforts, both in the acute and chronic phase [2]. Cognitive impairments are frequently reported in the chronic phase [3] and may have a negative impact on many aspects of a person’s life, including activities of daily living, work attainment, and quality of life [4, 5].

Computerized cognitive training has gained much attention as a restorative strategy [6, 7], targeting impaired processing speed, working memory and sustained attention [8, 9]. Restorative techniques aim to alleviate the specific impaired cognitive function through massed training trials [10]. These interventions build on the rationale that specific exercises within specific cognitive domains lead to generalized effects to other tasks than those that are trained [11]. CogMed, Lumiosity and BrainHQ are well-known computerized training programs [11], consisting of variations of verbal and visuospatial memory span tasks [12].

Studies involving computerized cognitive rehabilitation research suggest some efficacy [9], but several methodological issues have been raised, such as small number of participants, lack of control groups and differences in training dosages. The major concern related to computerized training interventions is however the lack of ecological validity, i.e. relevance to everyday life [13]. Outcome measures have often mimicked training content, making the generalizability of effects impossible to evaluate. One extensive literature review and one meta-analysis concluded that to the degree that computerized cognitive training had any effects, these were only short term and did not generalize to everyday functioning [8, 12].

There is a growing optimism regarding the usefulness of virtual reality (VR) in cognitive training intervention. This is due to its capacity for providing contextualized environments that resemble everyday situations, and the possibility to high training volume over short periods of time, in safe environments [6]. VR provides a non-invasive, immersive, computer-generated three-dimensional environment, in which the task difficulty can be adjusted and individualized. VR allows the user to interact with virtual objects, via sensorimotor channels in a head-mounted display, while muting outside stimuli [14]. Compared to technologies where stimuli are displayed on a two-dimensional computer screen, VR greatly increases the sense of presence, through its capacity to realistically convey visual and auditory information [15]. The experience of being in a virtual environment [16] provides the patient with rich and contextualized stimuli and tasks that may resemble everyday cognitive challenges, which may increase motivation and engagement, and potentially promote generalization of treatment effects [17].

The development of specialized VR systems for cognitive rehabilitation to ensure ecologically valid cognitive exercise tasks are called for [18, 19]. At present, few such options exist. In addition, specialized games developed for rehabilitation purposes often resemble cognitive tests and gamification aspects are less emphasized. Commercially available VR games, on the other hand, apply elements from gamification theory [20] that enhance motivation, such as clearly stated goals, salient rewards and adjustable difficulty levels, factors that are known to predict favorable outcomes following cognitive training [21]. As a result, research suggests that commercial video games may be as effective in improving attention as games created for rehabilitation [22].

A relatively recent review of VR interventions in cognitive rehabilitation after ABI identified only 13 studies [16]. While most of them concluded that VR was an effective method for training attention, memory, and problem-solving, scrutiny revealed severe methodological challenges. Only half of the studies involved immersive VR [23–28], while the rest used video games played on a screen. All studies utilizing immersive VR were published previous to the first generation of the VR systems that are in use today, rendering the technology non-comparable. Furthermore, small sample sizes were the norm and several studies were single case studies or lacked control groups [19]. Importantly, none investigated the effect of playing commercial VR games. There is thus a need for well-controlled randomized trials exploring the effectiveness of VR interventions in cognitive rehabilitation after ABI, and possible transfer effects to everyday activities [29].

Motivating effects of VR for healthy people are well established [30]. Our clinical experience, and the preliminary existing literature, indicates that most patients find it motivating to use VR for training purposes as well and tolerate it without major adverse effects. Cybersickness and fatigue are potential side effects of VR, particularly after extended use [31]. Cybersickness is similar to motion sickness, and it is not known if persons with ABI are more susceptible to these adverse effects, or if VR may exacerbate pre-existing symptoms in some individuals. To our knowledge, the only study reporting adverse effects of VR in an ABI population found that the technology was well tolerated by 18 of 21 patients [27], and that those who were not able to complete the training experienced “fatigue and frustration.” Furthermore, there are likely individual differences, both related to injury and other factors, when it comes to who might benefit from VR in rehabilitation. This has however not been systematically investigated.

### Aims and hypotheses

The primary aim of this study is:

- To evaluate the effect of playing a commercial VR game as a means of re-training of processing speed, working memory, and sustained attention in persons in the chronic phase of acquired brain injury.

Secondary aims are to explore:

- Transfer effects to everyday functioning.
- How ABI patients experience using VR.
- How fatigue, motivation and presence is affected by VR training.

We hypothesize that the intervention group will demonstrate improved processing speed, working memory, and sustained attention compared to an active control group.

We furthermore hypothesize to find generalization of VR treatment effects to improved everyday functioning. Finally, we anticipate that the majority of patients with ABI will tolerate the use of VR well, and find the method motivating, but that a minority will experience adverse effects.

## Methods

### Study design

**Fig. 1:**
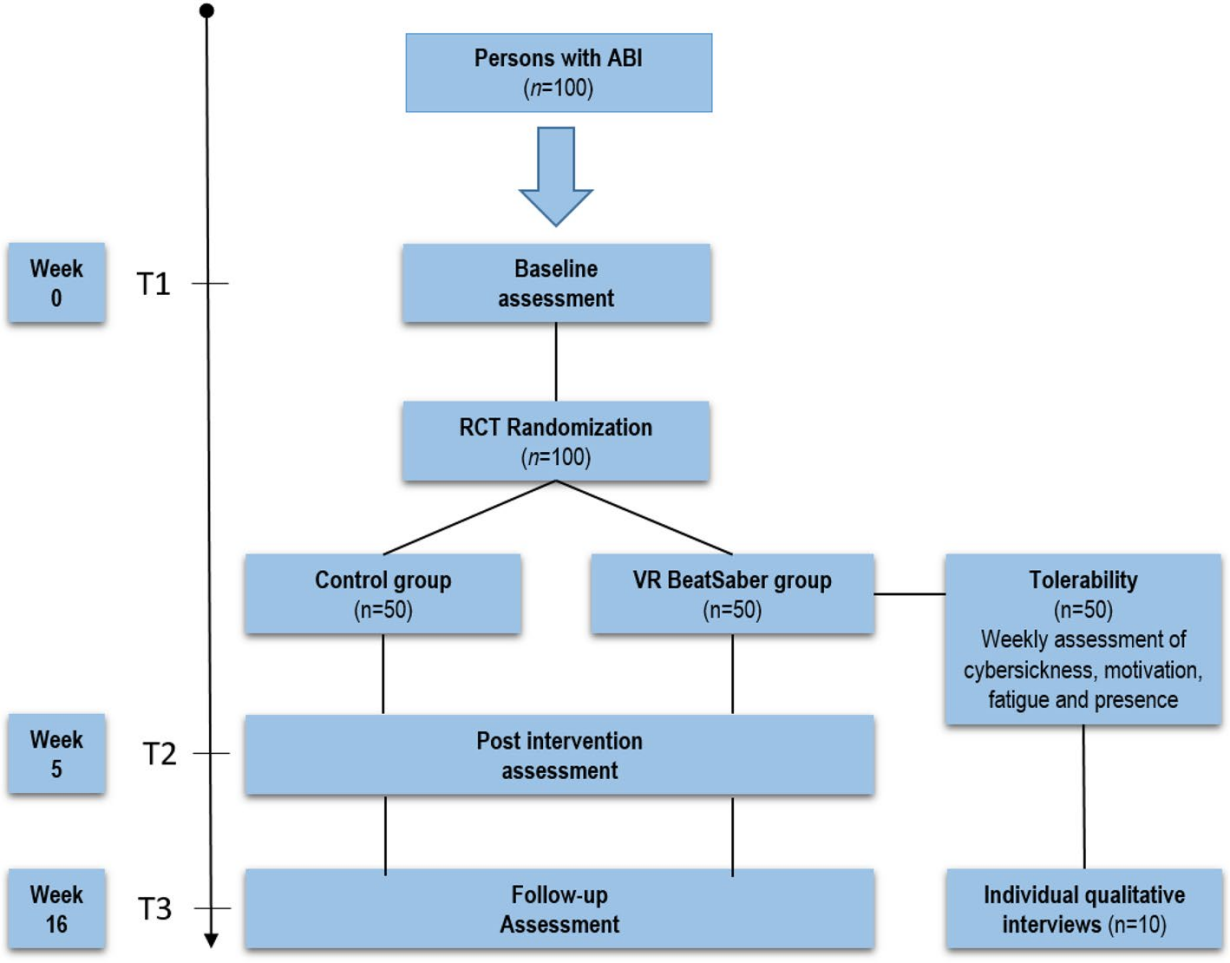
Overview of study design

The study will be conducted as a two parallel-group exploratory randomized controlled trial (RCT) with an active control group. Participants will be randomized into either VR training or a control group, after baseline assessment, with an allocation ratio at 1:1 (Fig. 1). During the intervention period, participants in both groups will be followed up with a ten-minute phone call once per week, with standardized questions to each of the study conditions. This aims to increase fidelity and adherence to protocol, and adverse events will be monitored. For the participants in the intervention group, the phone call will also include technical issues if needed, and assessment of sense of presence (Multi-Modal Presence Scale [32]) during the intervention. Participants in both groups will answer weekly questionnaires regarding motivation (Situational Motivation Scale [33]) and fatigue (Fatigue Severity Scale [34]).

The primary and secondary outcome measures will be re-administered at the end of the 5-week intervention period (T2) and 16 weeks (T3) after baseline. After T3, the first ten participants from the VR group will be asked to take part in individual qualitative interviews to gain in-depth understanding of their experience with the use of VR as a training method and participation in the intervention.

The RCT will follow the CONSORT statement [35] and is registered in ClinicalTrials.gov (NCT05443542) and Open Science Framework (osf.io/6gphy). When preparing this protocol, we used the SPIRIT reporting guidelines and the SPIRIT checklist is available as an additional file (Additional file 1).

### Study setting

**Fig. 2:**
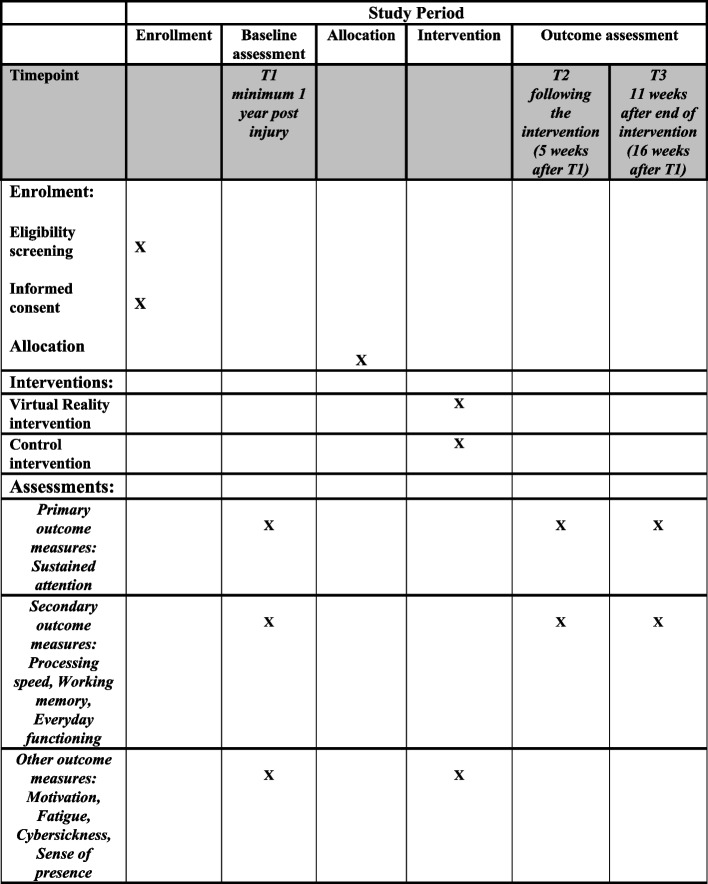
Standard protocol items: recommendations for intervention trials (SPIRIT)

T1 (week 0), T2 (week 5), and T3 (week 16) assessments will be performed at Sunnaas Rehabilitation Hospital, the largest rehabilitation hospital in Norway and the owner of the study. Participants will receive tutorials on the use of the VR technology at baseline, and the interventions will be carried out in the participant’s homes (Fig. 2).

### Participants

Patients with ABI who have been admitted to Sunnaas Rehabilitation Hospital will be considered for inclusion, and screened for eligibility. Eligible participants will receive a letter with an invitation to participate in the study. If participants have not responded to the letter within fourteen days, the project members will call them and ask whether they are interested in participating. A next of kin nominated by the patient will be included to obtain comparative information on the participants’ everyday functioning.

#### Eligibility criteria

Inclusion criteria:

- Patients with ABI (traumatic brain injury, cerebrovascular incidents, anoxia, encephalitis, and non-progressive brain tumors)
- At least 12 months post injury
- Aged 18–65 years
- Objective or subjective impairments of processing speed, working memory, or sustained attention
  ◦ Objectively determined via neuropsychological assessments of processing speed, working memory, and sustained attention documented in medical journal
  ◦ Subjectively determined through a screening interview that include open-ended questions about perceived everyday processing speed, attention and working memory. The interview also contains the two items for attention and processing speed in the Rivermead Concussion Symptoms Questionnaire.
- Participants need to:
  ◦ Be physically able to operate VR equipment
  ◦ Understand instructions in Norwegian or English
  ◦ Be able to provide informed consent

Exclusion criteria:

- Aphasia affecting the ability to understand instructions
- Apraxia affecting the ability to operate the VR equipment
- Visual neglect
- Photosensitive epilepsy
- Severe mental illness or active substance use disorder
- Comorbid neurological disorders

### Descriptive and outcome measures

All outcome measures will be administered at T1, T2, and T3. The primary outcome is sustained attention and will be measured by the coefficient of variation (CoV) on the Conners Continuous Performance Test 3rd (CPT 3) [36]. Secondary outcomes are CPT 3, Mean hit Reaction time, Weschler Adult Intelligence Scale IV, Backwards Digit Span and Digit Sequencing [37], and patient competency rating scale [38]. Psychometric properties for each measure are acceptable. See Table 1 for an overview of all primary and secondary outcome measures, citations for psychometric properties, respondents, and time points.

**Table 1:** Outcome measures, respondents and time points

*Outcome domain*	*Measure*	*Respondent*	*Time point*
**Sustained attention**	Conners Continuous Performance Test 3rd, coefficient of variation [39]	All participants	T1, T2, T3
**Processing speed**	Conners Continuous Performance Test 3rd, Mean hit Reaction time [39]	All participants	T1, T2, T3
**Working memory**	Weschler Adult Intelligence Scale IV, Backwards Digit Span and Digit Sequencing [40]	All participants	T1, T2, T3
**Everyday functioning**	Behavior Rated Inventory of Executive Functioning – Adult, working memory scale	All participants and next of kin	T1, T2, T3
	Patient Competency Rating Scale [38]	All participants	T1, T2, T3
**Motivation**	Situational Motivation Scale [41]	All participants	Weekly during intervention period
**Fatigue**	Fatigue severity scale [34]	All participants	Weekly during intervention period
**Cybersickness**	Simulator sickness questionnaire	Participants in VR group	Weekly during intervention period
**Sense of presence**	Multi-Modal Presence Scale [32]	Participants in VR group	Weekly during intervention period

At baseline (T1), neuropsychological test measures and questionnaires will be used for descriptive purposes. The following neuropsychological test battery will be applied: the Trail Making Test 4 (shifting) and subtest 3 of the Color Word Interference Test (inhibition) from the Delis-Kaplan Executive Function System test battery [42]. Abstract reasoning will be measured with Similarities and Matrices from WAIS IV [37]. Furthermore, we will include Coding from the WAIS IV as a measure of processing speed [37]. Immersive tendencies questionnaire will be administered at baseline to assess personality traits that may impact the level of experienced immersion [43].

Demographic data such as marital status, length and type of education and work status, will be gathered. In addition, injury-related data such as type and localization of injury will be collected.

### Randomization and allocation concealment

After the baseline assessment, the participants will be randomized into either the intervention or the control group. The randomization sequence will be generated electronically by an independent statistician, using block randomization [44] with a 1:1 ratio. Participants will be randomized after baseline assessment. The allocation sequence will be stored in a database that can only be accessed by the study’s principal investigator. Allocation will not be modified if the participant do not tolerate the intervention.

Blinding of participants and the researchers carrying out the intervention is impossible, but outcomes will be assessed by blinded assessors. Participants will be instructed to not reveal their group allocation to the outcome assessors. Data analysis will also be blinded as fake ID numbers will be assigned in the final database.

The personnel conducting the blinded assessments of the participants, will be trained by the research team to ensure data quality.

### Study interventions

#### Virtual reality

The experimental intervention is playing a commercially available VR game 30 min per day, for 5 weeks. The game selected is BeatSaber [45], which is a rhythm-based VR game that requires the player to react to colored blocks presented at the rhythm beats. As the game progresses the requirements of the game increases as the blocks are presented faster thus taxing both processing speed, sustained attention, and working memory. In addition to its cognitive requirements, the game was chosen due to availability on different platforms, which may ease implementation. It also has many users globally, securing the availability of the game in the future. The participants will be provided with a VR headset in order to conduct the VR training sessions at home. Throughout the intervention period they will have access to support, either by phone or video conference. Each 30-min session will be scheduled individually, with the instruction to use an alarm system to keep track of time. There are no existing clinical guidelines regarding recommended dosage and intensity of VR training; thus, the session length is based on clinical experience at SRH.

The VR game tracks the actual time each participant has spent playing the game during the intervention period. The amount of time spent playing will be recorded and used as a measure of intervention adherence.

#### Control group

An information booklet has been developed based on best practice evidence with information regarding compensatory strategies that participants can easily practice in their everyday lives. In addition to strategies, the booklet includes general information on topics that can affect cognitive functioning; national recommendation of physical exercise and physical activity, leisure activities (crossword puzzles, Sudoku, games and reading), sleep and nutrition. The control group will not be given a specific dosage; however, they will be encouraged to choose one or two topics to focus on during the intervention period. The control group will receive the same amount of therapist follow-up as the intervention group. The intervention group will not receive the information booklet.

At the end of the data collection period, the participants allocated to the control group will be offered to utilize VR as a method of training for a 5-week period. They will be given the possibility of the same amount of training and tutoring as the intervention group.

### Qualitative data collection

To investigate the participant’s experiences with VR as a method of cognitive training, semi-structured interviews will be conducted. An interview guide has been developed as recommended by Creswell [46], based on the acceptability framework developed by Sekhon and colleagues [47]. This includes questions regarding how participants feel about the intervention, the perceived amount of effort required to partake in the intervention, to what extent the participants understood the intervention, whether the participants perceived the intervention to result in its intended purpose, and the participant’s own belief that they can participate in the intervention. Interviews will be transcribed verbatim after each interview.

A pilot interview with one participant with previous experience with utilizing VR will be performed. After this an evaluation of the interview guide will be performed to assess whether amendments to the interview guide is needed.

### Technical solutions and data management

The intervention will be provided using the Oculus Quest 2 (Facebook Technologies, LLC). The Oculus Quest 2 is a wireless standalone VR headset that delivers the commercial VR game to the participants, creating the virtual environment through visual and auditory stimuli. The VR headset utilizes two motion-controlled hand controls that detect the hand movements of the participants in the virtual environment and provide haptic stimuli to the user. Oculus Quest 2 requires Meta accounts to set up the headset and to download and play the VR games. We will set up 20 Meta accounts, one for each headset.

Questionnaire data will be gathered using the University of Oslo’s solution for managing data, Services for Sensitive Data [48]. The TSD is an IT platform with a secure server that is approved for storing sensitive data for research purposes [48]. Questionnaires will be set up electronically using Nettskjema, which is a tool for designing and conducting online surveys [49].

All physical forms and test results will be kept in a locked cabinet. All data material will be recorded with the participant ID and will be de-identified. Only the researchers working on the project will have access to the list that connects participant IDs with names. Data will be stored electronically on a secure research server at SRH and will be deleted 5 years after the project period has ended. Only the research team will have access to the final trial dataset. Access to data is regulated by Norwegian laws regarding data protection and research ethics, and distribution of data is prohibited.

### Statistical analyses

Descriptive statistics will be performed to describe demographics, cognitive functioning and injury severity. Effect analysis of primary and secondary outcomes will be performed according to intention-to-treat analysis using data from all randomized participants, regardless of whether they complete the intervention. To determine changes in continuous outcome measures over time (T1, T2, and T3) between the groups, linear mixed-effects models will be used. Time and time-by-group interaction will be used as fixed effects and their interactions as indicators of the intervention effect. The main effect of group will be removed from the models to adjust for potential baseline differences [50]. In the same manner, we aim to explore associations between motivation, cybersickness, fatigue and sense of presence and interaction with time within the mixed-model framework. Estimated mean between group changes from baseline to T2 and T3 with 95% confidence intervals will be provided. For all outcomes, an alpha level of *p* = 0.05 will be applied.

Sub-group analyses of i.e. differences in duration since injury, age, and educational levels will be performed. Differences in continuous variables between groups will be tested using an independent sample *t*-test for normally distributed data, or by the Mann–Whitney *U* test for very skewed data. Linear regression analysis will be performed with registered adherence data as a possible predictor of treatment efficacy.

### Sample size and power calculation

Comparable studies to guide a potential anticipated effect for power calculation are lacking. However, similar studies investigating the outcome of attention utilizing computerized gaming interventions report within-group improvement scaling from one to two standard deviations [51]. In addition, a cross-sectional study investigating a similar population at two time points with the same outcome measures was also utilized [52]. The sample size was calculated based on the coefficient of variation (CoV) of CPT as the primary outcome. A mean difference in change in CoV of CPT of 3% between baseline and follow up between the groups was defined as a clinically important difference. With equal allocation to both treatment groups and with an SD of 5%, power of 80%, and a significance level of 5%, the sample size was calculated to be 45 patients in each group. Allowing for 10% drop out, we aim to include a total of 100 patients.

The sample for qualitative interviews will be selected using selective sampling, meaning selecting individuals who are knowledgeable about the intervention or phenomenon being studied [53]. In this study, the sample will be the first ten participants that finish the VR intervention period.

### Qualitative analyses

After all the interviews have been conducted, thematic analysis will be applied, following these six steps: (1) Organizing and preparing data, including transcribing and sorting field notes. (2) Reading through all the data, to obtain a general sense of what the information given by the participants. (3) Coding the data and organizing the material into segments of texts to start bring meaning to the information. (4) Separate the information into “descriptions” and “themes”. (5) Check for interrelating themes and descriptions. (6) Interpret the meaning of the themes and descriptions [54].

### Dissemination plan

The study plans for at least three publications in international peer-reviewed journals. We expect the novelty and innovative aspects of the study to allow for publication in high-impact journals within the field of neurology/rehabilitation and health innovation. Results will be disseminated in relevant expert forums, national meetings, conferences, and popular scientific journals and reports. Members of the local research group all have central positions within research, clinical management, and innovation at SRH. Implementation of study findings into clinical practice will thus be highly feasible. SRH plays a major role in spreading knowledge and contributing to the implementation of new knowledge throughout the rehabilitation sector, i.e., through hosting the South Eastern Regional Competency Centre for Rehabilitation. This will provide ample opportunity for national dissemination of results. The international collaborators will help to improve international dissemination of the results. We also expect study findings to be of interest for the general public.

## Discussion

The present RCT aims to investigate whether playing a VR game is an effective method of training processing speed, working memory, and sustained attention in persons with ABI. Furthermore, the study will explore if effects transfer to everyday functioning, and explore acceptability and tolerability of using VR for patients with ABI.

The research field regarding VR in cognitive rehabilitation is characterized by optimism, and a recent update to clinical guidelines of cognitive rehabilitation has listed VR as a method of cognitive training with level A for executive functions [55]. These recommendations are based on two reviews, including 9 and thirteen primary studies respectively [16, 56] and one cohort study [57]. However, closer scrutiny of the primary studies revealed that they have limitations. These limitations are in line with what Vlake and colleagues specify as common limitations in early VR studies: heterogeneous groups, lack of controls, and small samples [19]. In addition, the reviews pool together results from trials utilizing both immersive and non-immersive VR. A major shortcoming of available VR research today is that there are almost as many reviews as primary studies, which again may lead to an unqualified positive assessment of the existing evidence base. On this background, Vlake and colleagues call for large-scale studies with more homogenous groups and appropriate control conditions [19]. The present study thus represents a needed contribution to the field.

No studies have so far investigated the use of commercially available VR games in cognitive rehabilitation. However, BeatSaber has been suggested as an appropriate game to use in cognitive rehabilitation by Tao and colleagues [58]. Furthermore, based on clinical experience, our research team’s recognizes it as cognitively challenging, as it requires the player to react to stimuli with increasing speed and difficulty thus taxing both processing speed, sustained attention, and working memory.

Reviews call for further investigation of transferability of training effects after the use of VR into everyday functioning, larger and more homogenous sample sizes and utilizing proper control conditions [18, 29, 59]. To our knowledge, no studies have been performed with the methodological quality of the present study, and none has investigated whether the effects of VR training transfer into everyday functioning. This study will address all of these needs by including an adequate number of participants in reference to the sample size calculation, rigorous selection criteria and investigating everyday functioning both quantitatively and qualitatively.

There are many unknown factors with regard to the tolerability of VR use in the ABI population and persons with ABI. Thus, there is a need to investigate the acceptability and possible adverse events, i.e. tolerability, in more detail than previously evident in research performed on VR [19]. This study will qualitatively investigate the subjective experiences of using VR as a method of cognitive training in a subgroup of participants (*n* = 10). We will also explore the tolerability and acceptability of the intervention. Furthermore, all participants randomized to VR training will answer weekly questionnaires regarding adverse events, such as fatigue and cybersickness. Cybersickness is relatively rare while using VR, but it may be particularly relevant to assess in the ABI population as injuries to the brain may lead to symptoms that are comparable to symptoms of cybersickness. The qualitative data, in combination with the quantitative data collected during the intervention period, will provide valuable information regarding the usability, acceptability and tolerability of VR in the ABI population.

Many patients wish to continue cognitive training after being discharged from the hospital. If the present study shows VR to be effective, the intervention may provide an opportunity for persons with ABI to continue to do cognitive training after post-acute rehabilitation.

### Limitations

There are some limitations to the present study. Blinding of the participants and the researchers who conduct the intervention is impossible because the researchers have to give tutoring in the VR game to those who are randomized to the intervention group and perform follow-up conversations with questions corresponding to their group allocation. Because the participants will be aware of their group allocation, the management of the expectation of efficacy of the intervention is difficult, which could possibly affect their responses on subjectively reported outcome measures. However, the primary outcome measure is a neuropsychological measure of attention with estimated low expectancy effects.

It was difficult to find a comparator for VR differing from the intervention only in the aspects we want to investigate. Placebo VR, such as passive virtual experiences or VR videos, was deemed inappropriate since we are also exploring VR technology in itself. The control condition was set to be what is considered best practice of cognitive rehabilitation in the chronic phase, utilizing compensatory strategies and cognitive training in other everyday activities. Since these activities are close to the recommendations that are given to patients when discharged from hospital, it is considered by the project group to be as close to an active treatment condition as possible. Both the intervention and control group receives the same follow-up as the intervention group.

When conducting sample size calculation there were a lack of similar studies with appropriate methodology and adequate quality for estimation of expected effect size. This made sample size calculation challenging, but comparable studies utilizing other forms of cognitive training were used [52, 60].

Adherence will be challenging to monitor before the VR headset is returned at T2, since the intervention will be performed in the participants’ home. One of the researchers in the study will perform weekly follow-up conversations during the intervention period, where one of the questions relates to adherence.

Despite possible limitations of the current trial, the described design and methods to investigate the effectiveness of VR as a method of cognitive training are considered appropriate and will result in new information. This will add to the knowledge base for new intervention methods in rehabilitation of processing speed, working memory, and sustained attention in persons with ABI.

## Trial status

Recruitment for the RCT began in November 2022 and will continue until target sample size has been reached, estimated April 2024.

### Supplementary Information

Supplementary Material 1.
